# Effect of a Brief Sensitization Session on Tobacco Control Awareness Among Healthcare Professionals Across India: A Pre– and Post-intervention Evaluation

**DOI:** 10.7759/cureus.86422

**Published:** 2025-06-20

**Authors:** Saurabh Varshney, G. Jahnavi, Bijit Biswas, Arshad Ayub, Venkata Lakshmi Narasimha, Santanu Nath, Sudip Bhattacharya, Vinayagamoorthy Venugopal, Benazir Alam, Ujjwal Kumar, Niwedita Jha

**Affiliations:** 1 Otolaryngology, All India Institute of Medical Sciences, Deoghar, IND; 2 Community and Family Medicine, All India Institute of Medical Sciences, Deoghar, IND; 3 Psychiatry, All India Institute of Medical Sciences, Deoghar, IND; 4 Vital Strategies Tobacco Control Project, All India Institute of Medical Sciences, Deoghar, IND

**Keywords:** cotpa, healthcare professionals, sensitization, tobacco control, who-fctc

## Abstract

Background: Awareness of India’s tobacco control laws, including the Cigarettes and Other Tobacco Products Act (COTPA) and the WHO Framework Convention on Tobacco Control (FCTC), remains limited among healthcare professionals. This study evaluated the effect of a brief sensitization session on improving awareness across key tobacco control domains.

Methods: A multicity pre-post interventional study was conducted at 22 academic conferences (17 offline, five virtual) across 14 Indian cities between May 2023 and April 2025. A 30-minute standardized session covered COTPA provisions, tobacco industry interference (TII), WHO FCTC Article 5.3, and state-level COTPA implementation. A total of 1,233 healthcare professionals completed both pre- and post-intervention assessments. Data were analyzed using McNemar’s test, Wilcoxon signed-rank test, and Kruskal-Wallis test with post hoc comparisons.

Results: Significant improvements were observed in all four domains (p < 0.001 each). Awareness of COTPA increased from 533 (43.2%) to 1.040 (84.3%); TII from 596 (48.3%) to 1193 (96.8%); WHO FCTC Article 5.3 from 348 (28.2%) to 1117 (90.6%); and awareness of state-level implementation from 217 (17.6%) to 530 (43.0%). The median composite score rose from one (interquartile range (IQR): 0 to two) to three (IQR: three to four), with a median gain of two points (Wilcoxon W = 450775, p < 0.001; rank biserial correlation = 1.00). Score gains were significantly higher among medical faculty and participants from northern, eastern, and southern India (p < 0.001).

Conclusion: A brief, structured sensitization session significantly enhanced awareness of tobacco control policies among healthcare professionals and can support capacity-building efforts in India.

## Introduction

Tobacco consumption continues to pose a significant threat to public health worldwide, causing more than eight million deaths annually, with India alone accounting for over 1.3 million of these deaths each year [1-3]. Despite strong policy initiatives, tobacco use remains widespread in India, where nearly one in three adults consumes tobacco in some form, either smoked or smokeless [4].

Healthcare professionals, expected to counsel and model cessation behaviors, are not immune to this issue. Systematic reviews have shown that the prevalence of tobacco use among healthcare workers ranges between 2% and 37% globally, with Indian estimates typically falling between 2% and 43.4%, depending on cadre, gender, and workplace culture [5-8]. This continued use not only affects their health but also diminishes their effectiveness as tobacco control advocates.

To combat this, India enacted the Cigarettes and Other Tobacco Products Act (COTPA), 2003, which prohibits smoking in public places, bans direct and indirect advertising, and mandates graphic health warnings on packaging [9,10]. Additionally, under the WHO Framework Convention on Tobacco Control (FCTC), Article 5.3 specifically directs governments and institutions to shield public health policies from tobacco industry interference (TII) by promoting transparency, rejecting partnerships, and limiting engagement with tobacco-linked entities [11,12]. Nonetheless, awareness and understanding of these provisions among healthcare professionals remain suboptimal, which may hinder effective implementation [6,13,14].

Academic health institutions, including medical and nursing colleges, are uniquely positioned to lead tobacco control efforts. They are expected to cultivate tobacco-free environments, sensitize students and staff to anti-tobacco laws and global guidelines, and actively resist TII through informed policies and practice [12,15,16]. Evidence suggests that brief, targeted sensitization interventions, when incorporated into academic or professional settings, can significantly enhance awareness and readiness to act on tobacco control measures [17,18].

Against this backdrop, the present study sought to assess the impact of a short, structured sensitization session on improving tobacco control awareness among healthcare professionals across India. Specifically, it evaluated awareness in four critical areas: familiarity with COTPA, understanding of TII, knowledge of WHO FCTC Article 5.3, and awareness of the number of Indian states implementing key COTPA provisions.

## Materials and methods

Study design and setting

This multicity pre-post interventional study was conducted across 14 cities in India from May 2023 to April 2025. The intervention was delivered during 17 offline academic conferences, three held in Deoghar, two in Bengaluru, and one each in the remaining cities, representing five state-level, 10 national-level, one regional, and one international event. To enhance reach and maintain consistency, five additional virtual conferences conducted under the same project framework were included. All sessions, both physical and virtual, followed a standardized content structure to ensure uniform delivery.

Participants and sample size

The study population comprised healthcare professionals, including faculty members (medical, dental, and nursing), senior residents, postgraduate trainees or junior residents, nursing officers, and other clinical staff attending the conferences. Participation in the study was entirely voluntary. Only individuals who provided informed consent and completed both pre- and post-intervention assessments were included in the final analysis. The required sample size was estimated using Statulator (University of Sydney, Australia), assuming a minimum detectable effect size of 0.1, 1% precision, and 80% statistical power, resulting in a sample size of 1,172. Ultimately, 1,357 participants completed the pre-test, and 1,233 provided complete paired responses, which were included in the final analysis.

Intervention and procedure

Formal requests were submitted to organizers and parent associations of 32 academic conferences, clearly stating that the sensitization session would be delivered without any financial support or sponsorship. Of these, 17 conferences agreed to include a 30-minute session on tobacco control within their scientific agenda. The sessions, conducted by the investigators, aimed to enhance awareness of national and international tobacco control frameworks and highlight the institutional responsibilities of healthcare professionals in countering TII. A standardized format was followed across all sessions, covering three core components: key provisions of the COTPA, the WHO FCTC with emphasis on Article 5.3, and the proactive role of medical institutions in resisting TII. The content was delivered through structured presentations with interactive elements. Participants who completed both pre- and post-intervention assessments received an e-certificate acknowledging their participation in the survey.

Data collection and outcome measures

A structured questionnaire was administered before and after each session via Google Forms (Google Inc., Mountain View, CA) (Appendix A). The form began with a brief introduction to the study and embedded informed consent. Only those who consented were allowed to proceed. Responses were non-anonymous and matched using unique identifiers (i.e., mobile number) to allow pre-post comparison. The questionnaire assessed awareness across four items: knowledge of COTPA, awareness of tobacco industry interference, familiarity with WHO FCTC Article 5.3, and knowledge of the number of Indian states implementing COTPA provisions, as per the most recent Global Adult Tobacco Survey (GATS). Correct or affirmative responses were scored as one, while incorrect or "don't know" responses were scored as 0. A composite awareness score ranging from 0 to four was computed for each participant. Internal consistency of the four-item scale was assessed using Cronbach’s alpha, which yielded values of 0.735 pre-intervention and 0.751 post intervention, indicating acceptable reliability. The primary outcome was the change in composite awareness score following the intervention.

Statistical analysis

All statistical analyses were conducted using Jamovi version 2.3.28. Categorical variables were summarized using frequencies and percentages, and non-normally distributed continuous variables were expressed as medians with interquartile ranges (IQR). McNemar’s chi-square test was used to assess item-wise changes in awareness before and after the intervention. The Wilcoxon signed-rank test was applied to compare composite awareness scores pre- and post-intervention, and the effect size was reported as a rank biserial correlation. Associations between awareness score changes and participant characteristics such as occupation and region of residence were analyzed using the Kruskal-Wallis test. For statistically significant associations, Dunn’s post hoc test with Bonferroni correction was used for pairwise comparisons. A p-value of <0.05 was considered statistically significant.

## Results

**Figure 1:**
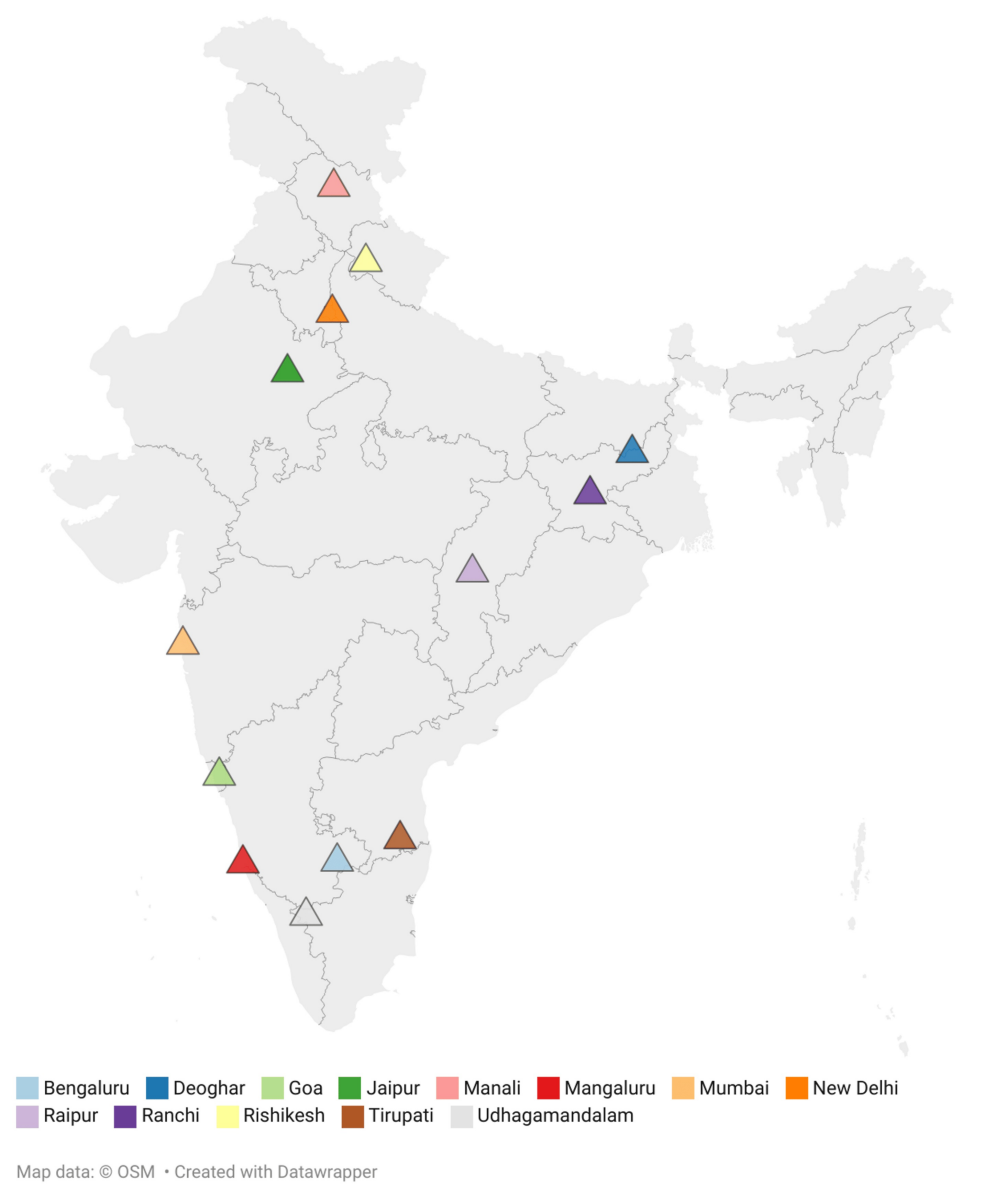
Geographical distribution of event locations across India used for tobacco control advocacy: (n=14) Map data: ©OSM, Created with Datawrapper (Datawrapper GmbH, Berlin, Germany)

**Figure 2:**
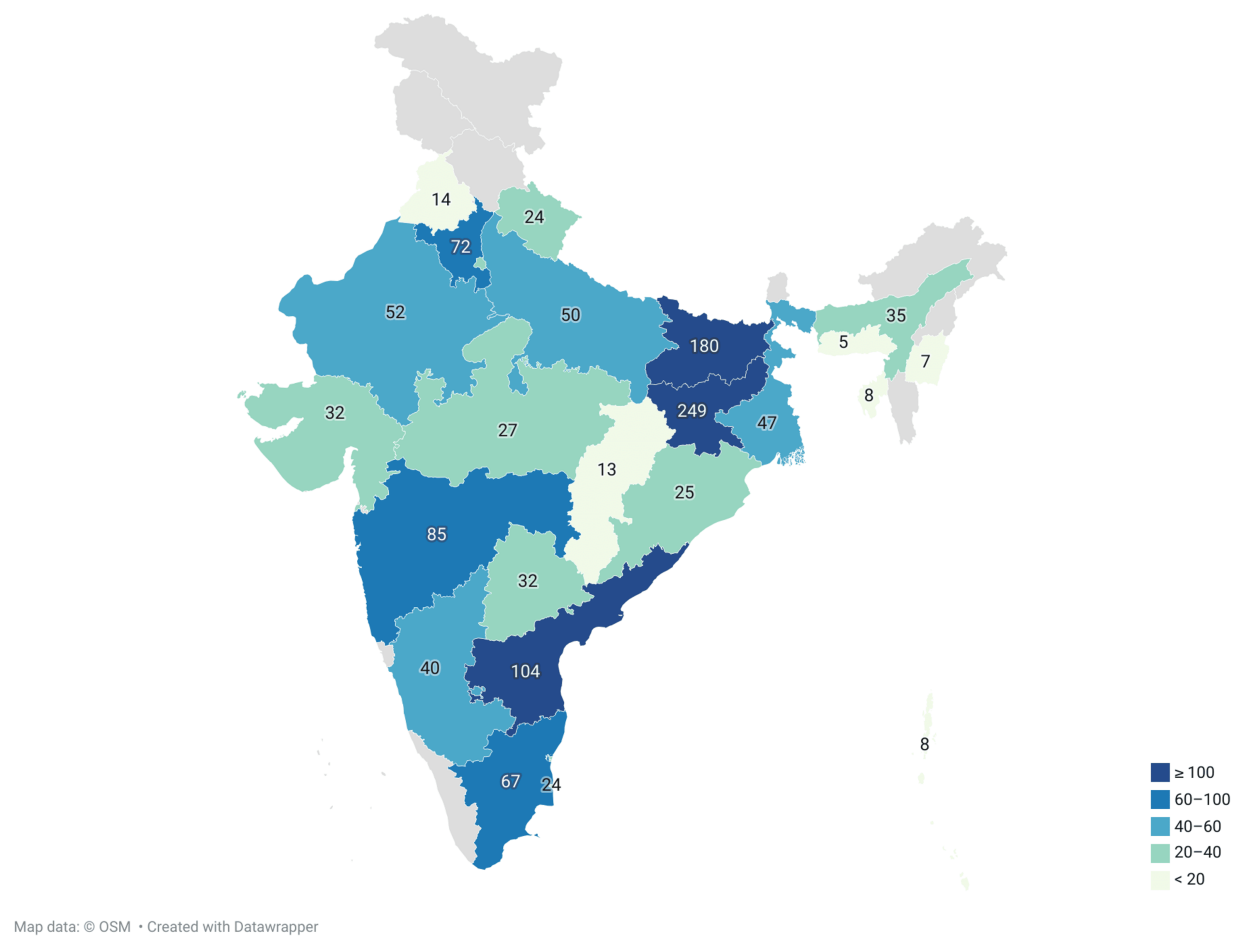
Geographic distribution of study participants across India, based on their current state of residence at the time of survey participation: (n=1233) Map data: ©OSM, Created with Datawrapper (Datawrapper GmbH, Berlin, Germany)

Among the conferences included in the study, the highest number were conducted in southern India (five; 22.7%), followed by eastern India (four; 18.2%), northern India (three; 13.6%), western India (two; 9.1%), and central India (one; 4.5%). Additionally, five conferences (22.7%) were conducted virtually, ensuring pan-India participation across diverse healthcare institutions (Figure 1). In terms of participant representation, the highest proportion was from Jharkhand (249; 20.2%), followed by Bihar (180; 14.6%), Andhra Pradesh (104; 8.4%), and Maharashtra (85; 6.9%). When participants from Jharkhand and Bihar (n = 804) were excluded, most were from southern India (275; 34.2%), followed by northern (193; 24.0%), western (169; 21.0%), eastern (127; 15.8%), and central India (40; 4.9%) (Figure 2).

**Figure 3:**
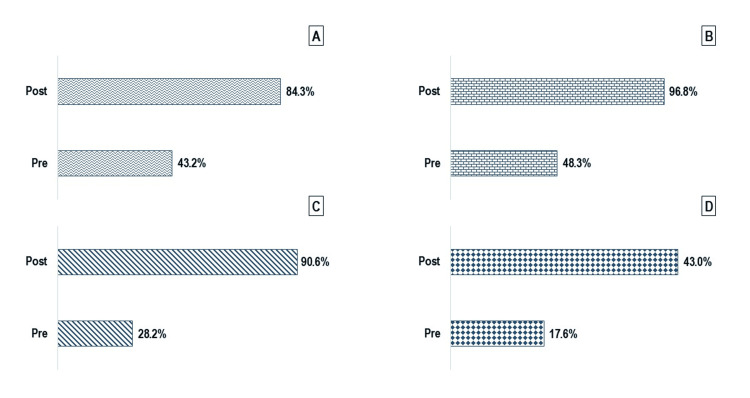
Item-wise distribution of study participants’ knowledge on tobacco control before and after the intervention: (n = 1233) A: heard about the Cigarette and Other Tobacco Products Act (COTPA); B: heard about tobacco industry interference (TII); C: heard about the World Health Organization Framework Convention for Tobacco Control (WHO FCTC) ARTICLE 5.3; knew about the number of states implementing COTPA as per the latest Global Adult ’Tobacco Survey (GATS). Data represented as percentages and compared by McNemar’s chi-square test with p-values of <0.05 were considered significant.

**Figure 4:**
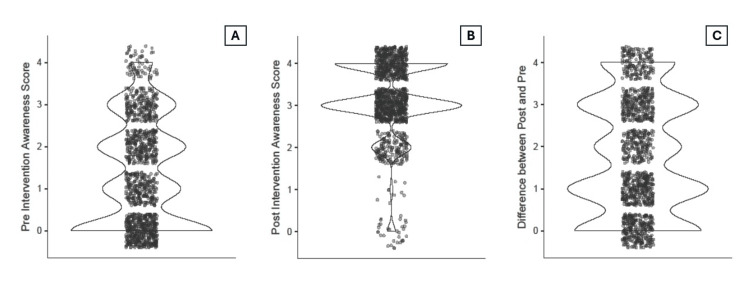
A violin plot showing the distribution of study participants’ knowledge scores on tobacco control before and after the intervention, including the difference between pre- and post-intervention scores: (n=1233) A: Pre-intervention awareness score; B: Post-intervention awareness score; C: Difference between post- and pre-intervention awareness scores

Among the 1,233 participants with paired responses, statistically significant improvements were observed across all four tobacco control awareness items following the sensitization session (p < 0.001 for each; Figure 3). Awareness of the COTPA increased from 533 (43.2%) pre-intervention to 1,040 (84.3%) post intervention. Awareness of TII rose from 596 (48.3%) to 1,193 (96.8%), while familiarity with the WHO FCTC Article 5.3 improved from 348 (28.2%) to 1,117 (90.6%). Knowledge of the number of Indian states implementing key COTPA provisions, as reported in the latest GATS, increased from 217 (17.6%) to 530 (43.0%). The median composite awareness score rose from 1 (IQR: 0 to two) pre-intervention to three (IQR: three to four) post intervention, with a median change of two points (IQR: one to three). This improvement was statistically significant based on the Wilcoxon signed-rank test (W = 450775, p < 0.001), and the rank biserial correlation was 1.00, indicating a very large effect size (Figure 4).

**Figure 5:**
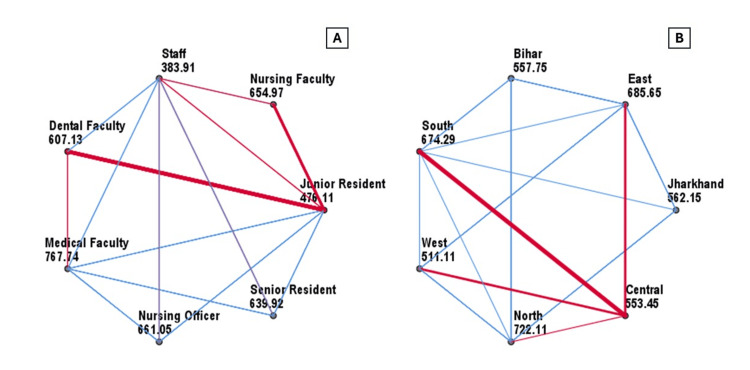
Pairwise comparison of study participants based on occupation, current region of residence, and effect of brief sensitization session on tobacco control (n=1233) A: Change in post-intervention awareness score by occupation; B: Change in post-intervention awareness score by current region of residence. Statistical analysis was performed using the Kruskal–Wallis test.

Further analysis revealed that occupation and region of residence were significantly associated with changes in awareness scores post intervention (both p < 0.001). Post hoc comparisons showed that medical faculty had significantly higher awareness gains compared to staff (mean rank difference = 383.8, p < 0.001), junior residents (291.6, p < 0.001), senior residents (127.8, p = 0.001), nursing officers (106.7, p = 0.001), and dental faculty (160.6, p = 0.012). Senior residents and nursing officers had higher gains than junior residents (163.8 and 184.9, respectively; both p < 0.001). Dental faculty and junior residents also scored higher than staff (223.2, p = 0.039, and 92.2, p = 0.024, respectively). Regional comparisons showed participants from eastern India had higher gains than those from western India (174.5, p < 0.001) and Bihar (127.9, p = 0.032). Similarly, participants from southern India had higher gains than those from western India (163.2, p < 0.001), Bihar (116.5, p = 0.010), and Jharkhand (112.1, p = 0.005). Participants from northern India showed significantly greater gains than those from western India (211.0, p < 0.001), Bihar (164.4, p < 0.001), Jharkhand (160.0, p < 0.001), and central India (168.7, p = 0.005) (Figure 5).

## Discussion

This multicity pre-post study evaluated the effectiveness of a brief sensitization session on improving tobacco control awareness among healthcare professionals in India. Before the intervention, only about every third participant was aware of the WHO FCTC, and just two out of five had heard of COTPA. Awareness of tobacco industry interference was slightly higher but still limited. Knowledge of state-level implementation of tobacco laws was the poorest, with only every fifth participants aware. Following the session, awareness rose markedly; about four out of five participants became familiar with COTPA, and almost all could identify tobacco industry interference and WHO FCTC Article 5.3. Awareness of state-level implementation also improved significantly, with nearly every second participant reporting familiarity. These findings highlight the effectiveness of even a short, structured educational session in bridging key policy-related knowledge gaps among healthcare professionals.

At baseline, awareness of tobacco control policies among healthcare professionals was uneven, with several key elements significantly underrecognized. In our study, 43.2% of participants had heard of the COTPA, a figure notably higher than the 16.1% reported among slum communities in Delhi by Sharma et al. [14], yet similar to the 44.1% awareness of age-related tobacco sale restrictions among educated youth in Haryana reported by Verma et al. [13]. This suggests that while healthcare professionals may benefit from greater exposure, it does not uniformly translate into comprehensive policy understanding. In comparison, Biswas et al. [6] reported substantially higher awareness, ranging from 51.9% to 91.4%, regarding specific provisions of COTPA among healthcare students, professionals, and staff in medical and dental colleges of Bihar and Jharkhand. In our study, less than half (48.3%) of participants were aware of tobacco industry interference, and only 28.2% had heard of Article 5.3 of the WHO FCTC, which addresses protection of health policies from industry influence. Particularly striking was the low awareness (17.6%) regarding the number of Indian states implementing COTPA provisions, highlighting a substantial gap in knowledge about enforcement and ground-level policy application.

In the present study, tobacco control awareness improved substantially following the intervention, with a rank biserial correlation of 1.00, denoting a very large effect size. These results align with findings by Paul et al. [18], who reported significant knowledge gains on COTPA among non-teaching staff in a tertiary care hospital in Uttar Pradesh after structured training. This reinforces the effectiveness of brief, focused interventions in enhancing legal awareness across healthcare settings. Furthermore, changes in awareness scores were significantly associated with participants’ occupation and region of residence (both p < 0.001). Medical faculty demonstrated the greatest improvement, significantly outperforming staff, residents, nursing officers, and dental faculty, suggesting greater receptivity to policy-related content. Senior residents and nursing officers had higher gains than junior residents, possibly reflecting increased clinical engagement. Regionally, participants from eastern, southern, and northern India achieved significantly greater improvements compared to those from western India, Bihar, Jharkhand, and central India, highlighting disparities in baseline awareness or institutional emphasis. These findings support the need for tailored, cadre-specific, and region-sensitive strategies to maximize the reach and effectiveness of such sensitization efforts.

This study has certain limitations. First, the use of a self-administered online questionnaire may have introduced response bias; however, linking responses pre-post using unique identifiers helped ensure internal validity. Second, participants were primarily from academic conference settings, which may limit generalizability; nonetheless, the diversity of locations and professions adds representativeness. Third, awareness was measured immediately post intervention without follow-up, so long-term retention could not be assessed, though the intervention's structured delivery likely reinforced key messages. Lastly, the questionnaire used a limited number of items, yet it focused on core domains aligned with national and global tobacco control priorities.

## Conclusions

This study showed that a brief sensitization session delivered through academic conferences significantly improved tobacco control awareness among healthcare professionals. Awareness of COTPA increased from approximately two out of five participants before the intervention to four out of five after. Awareness of tobacco industry interference rose from just under half to almost all participants. Familiarity with WHO FCTC Article 5.3 increased from about one in three to nearly all, while knowledge of state-level implementation of tobacco laws improved from every fifth to over two out of five participants. These findings confirm that short, structured sessions can lead to measurable improvements in awareness across multiple domains of tobacco control.

## Appendices

Appendix A

Brief Session on Tobacco Control Awareness

Link: https://drive.google.com/file/d/1eextJtQk-t_45t_f2Z9A1E2TT48XeTL4/view?usp=sharing 

**Table 1:** Tobacco Control Awareness Assessment Tool

Item	Question	Responses	Score
K1	Have you ever heard of the Cigarettes and Other Tobacco Products Act (COTPA)?	Yes	1
		No	0
K2	Are you aware of the concept of Tobacco Industry Interference (TII)?	Yes	1
		No	0
K3	Have you heard of Article 5.3 under the World Health Organization Framework Convention on Tobacco Control (WHO-FCTC)?	Yes	1
		No	0
K4	As per the latest Global Adult Tobacco Survey (GATS), how many Indian states or union territories have implemented COTPA? (Open-ended)	28	1
		Other Responses	0
